# Systems biology comprehensive analysis on breast cancer for identification of key gene modules and genes associated with TNM-based clinical stages

**DOI:** 10.1038/s41598-020-67643-w

**Published:** 2020-07-02

**Authors:** Elham Amjad, Solmaz Asnaashari, Babak Sokouti, Siavoush Dastmalchi

**Affiliations:** 1 Biotechnology Research Center, Tabriz University of Medical Sciences, Tabriz, Iran; 2 School of Pharmacy, Tabriz University of Medical Sciences, Tabriz, Iran

**Keywords:** Computational biology and bioinformatics, Systems biology

## Abstract

Breast cancer (BC), as one of the leading causes of death among women, comprises several subtypes with controversial and poor prognosis. Considering the TNM (tumor, lymph node, metastasis) based classification for staging of breast cancer, it is essential to diagnose the disease at early stages. The present study aims to take advantage of the systems biology approach on genome wide gene expression profiling datasets to identify the potential biomarkers involved at stage I, stage II, stage III, and stage IV as well as in the integrated group. Three HER2-negative breast cancer microarray datasets were retrieved from the GEO database, including normal, stage I, stage II, stage III, and stage IV samples. Additionally, one dataset was also extracted to test the developed predictive models trained on the three datasets. The analysis of gene expression profiles to identify differentially expressed genes (DEGs) was performed after preprocessing and normalization of data. Then, statistically significant prioritized DEGs were used to construct protein–protein interaction networks for the stages for module analysis and biomarker identification. Furthermore, the prioritized DEGs were used to determine the involved GO enrichment and KEGG signaling pathways at various stages of the breast cancer. The recurrence survival rate analysis of the identified gene biomarkers was conducted based on Kaplan–Meier methodology. Furthermore, the identified genes were validated not only by using several classification models but also through screening the experimental literature reports on the target genes. Fourteen (21 genes), nine (17 genes), eight (10 genes), four (7 genes), and six (8 genes) gene modules (total of 53 unique genes out of 63 genes with involving those with the same connectivity degree) were identified for stage I, stage II, stage III, stage IV, and the integrated group. Moreover, SMC4, FN1, FOS, JUN, and KIF11 and RACGAP1 genes with the highest connectivity degrees were in module 1 for abovementioned stages, respectively. The biological processes, cellular components, and molecular functions were demonstrated for outcomes of GO analysis and KEGG pathway assessment. Additionally, the Kaplan–Meier analysis revealed that 33 genes were found to be significant while considering the recurrence-free survival rate as an alternative to overall survival rate. Furthermore, the machine learning calcification models show good performance on the determined biomarkers. Moreover, the literature reports have confirmed all of the identified gene biomarkers for breast cancer. According to the literature evidence, the identified hub genes are highly correlated with HER2-negative breast cancer. The 53-mRNA signature might be a potential gene set for TNM based stages as well as possible therapeutics with potentially good performance in predicting and managing recurrence-free survival rates at stages I, II, III, and IV as well as in the integrated group. Moreover, the identified genes for the TNM-based stages can also be used as mRNA profile signatures to determine the current stage of the breast cancer.

## Introduction

Breast cancer (BC) is one of the most common health threatening problems among women in the world, leading to death of those patients with BC^1^. It has been reported in 2019 that the incidence and mortality of breast cancer worldwide are 24.2% and 15.0%, respectively, deserving more attention from healthcare systems and policy-makers^1^. To clinically classify the status of breast cancer, the American Joint Committee on Cancer (AJCC) has announced eight editions on the Tumor-Node-Metastasis (TNM)-based staging of breast cancer, specifically for treatment and prognosis^2,3^. Since more than 50% of the affected patients were died, increasing the survival rate of these patients is highly important by determining the stage of the disease. The earlier the identification of the stage, the more superior the survival rate. To increase the therapeutic efficiency and consider the molecular portrait differences in BC along with their different clinical outcomes^4^, breast cancer can be classified into six main subtypes, including normal-like, luminal A, luminal B, HER2-positive, basal-like, and claudin-low^5^; the classification has also been confirmed by the Cancer Genome Atlas (TCGA) program^6^.

It has been frequently reported that the human epidermal growth factor receptor (HER) family (i.e., HER-1, HER-2, HER-3, and HER-4) plays a pivotal role in various cancers^7^. Among them, HER-2 (known as HER-2/neu gene), as an oncogene with 1,255 amino acids and 185kD transmembrane glycoprotein with tyrosine kinase activity, is located at chromosome 17^7,8^. Moreover, HER-2/neu gene makes breast cancer classified as HER2-positive and HER2-negative^9^. In 15–30% of patients with invasive breast carcinomas, an overexpression or amplification of HER2 has been identified^7,10^.

It is worth mentioning that is not effective for HER2-negative. Although, endocrine therapy is the target of chemotherapy, there are no successful reports for survival rates of these types of patients in the literature^11^. Moreover, several traditional diagnostic approaches such as mammography, magnetic resonance imaging (MRI), ultrasound, computerized tomography (CT), positron emission tomography (PET), and biopsy have been studied in breast cancer diagnosis^12^.

Nowadays, molecular biomarkers have been proposed to provide more efficiency in the prognosis and diagnosis of cancers in deficiency of traditional cancer tests. Additionally, the biomarkers are now regularly utilized to better understand the development of the tumors^13^. Hence, owing to the large number of stored microarray gene expression profiles by several genomics laboratories in the most publicly available database websites such as National Center for Biotechnology Information (NCBI), their analyses by various bioinformatics and systems biology analyses are essential^4^. Finally, these biomarkers will be helpful in personalizing the treatments for each patient with their special stage of the disease^4^. Considering the HER2-targeted therapy, there are still no predictive biomarkers validated for the prognosis and diagnosis of the stages of breast cancer^14,15^.

Consequently, the aim of the current study is to identify the potential biomarkers in breast cancer at stages I, II, III, IV as well as in the integrated group simultaneously regarded as one. To reach this aim, three microarray gene expression profiling datasets have been included to identify the differentially expressed genes (DEGs). By prioritizing those DEGs, their cellular and molecular functions will be further analyzed. Then, the involved GO (Gene Ontology) and KEGG (Kyoto Encyclopedia of Genes and Genomes) signaling pathways will be studied. Moreover, the protein–protein interaction network for all stages are developed based on the STRING database, and the significant hub genes are identified by clustering algorithm from which the gene biomarkers will later be determined based on their higher connectivity degrees. Finally, the Kaplan–Meier analysis tool was used to assess recurrence-free survival rates of the identified gene biomarkers.

## Materials and methods

**Figure 1:**
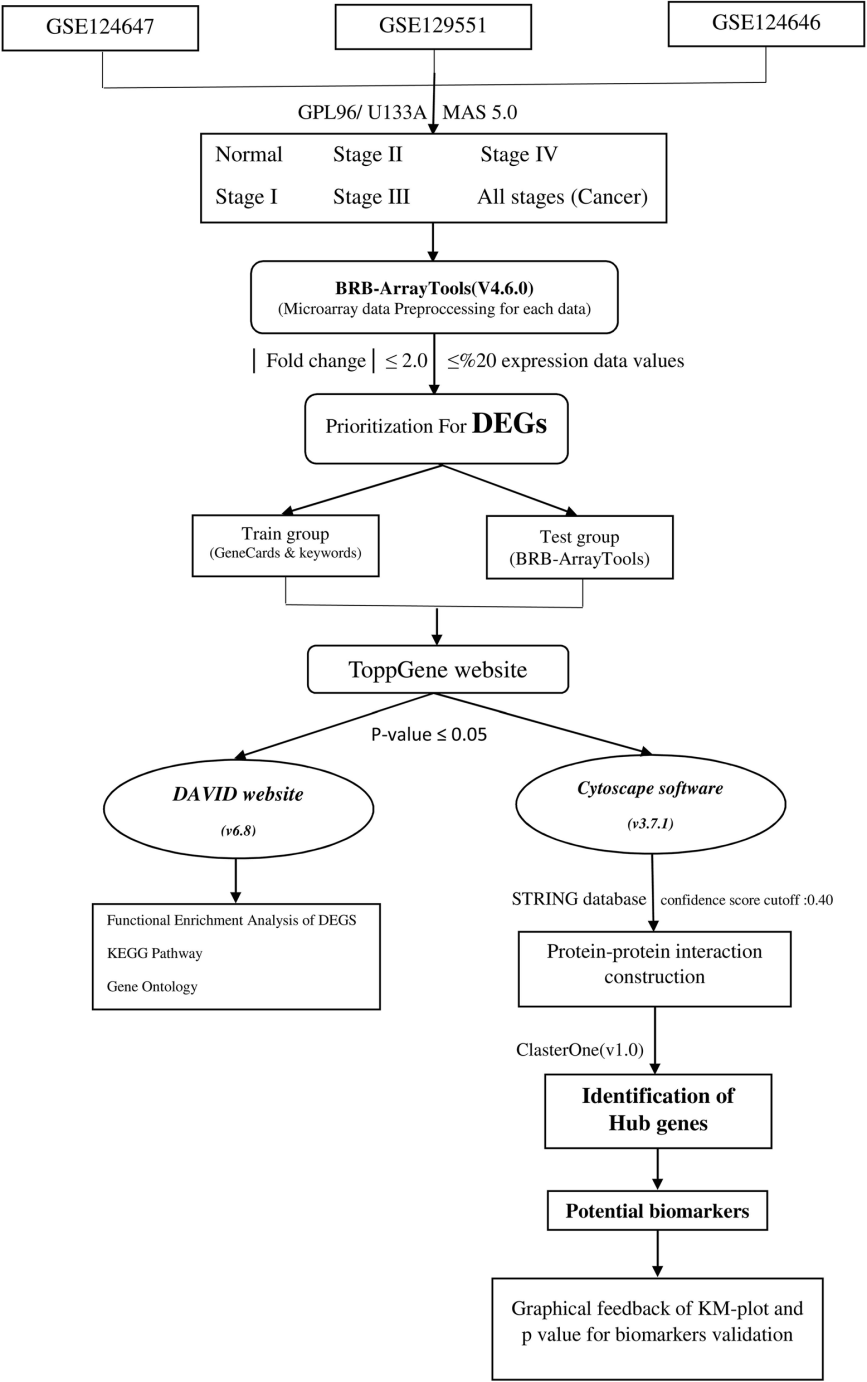
Flowchart of the current research approach step by step to achieve the final validated gene biomarkers in terms of recurrence free survival in HER2-negative breast cancer.

Figure 1 presents the summarization of the flowchart diagram of the approach to satisfy the research question.

### Data sources

All the datasets used in this study were retrieved from the NCBI GEO database (i.e., https://www.ncbi.nlm.nih.gov/geo/). The platform and file type of the breast cancer microarray datasets were GPL96 [HG-U133A] Affymetrix Human Genome U133A Array and CEL files, respectively. To cover the aim of this study, GSE124647, GSE129551, and GSE124646 were used as train set including 140 biopsy samples from metastatic patients with stage IV breast cancer, 147 samples from patients with stages I, II, III, and IV breast cancer, and 10 normal samples (0 percent cancer) out of 100 samples, respectively. Moreover, GSE15852 (i.e., includes 43 normal, 8 grade 1 ~ stage I, 23 grade 2 ~ stage II, and 12 grade 3 ~ stage III samples) was used as a test set for external validation.

### Data preprocessing and identification of differentially expressed genes (DEGs)

The BRB-ArrayTools (v4.6.0, stable version), an excel graphical user interface (GUI) for communicating with R (v 3.5.1) programming environment developed by Dr. Richard Simon and the BRB-ArrayTools Development Team, was used for all stages of preprocessing (i.e., data import, data filtering, and normalization), gene annotation using “hthgu133a.db” R annotation package^16^ and identification of DEGs. During the data import phase, Microarray Suite version 5.0 (MAS 5.0) algorithm was utilized, and then spot filtering, quantile normalization, and gene filtering (gene exclusion criteria of fold change ≤ 2 with expression data values less than %20) were carried out. Next, class comparison between groups of arrays in terms of their label classification was performed to identify the differentially expressed genes (DEGs) by enabling the two options, including univariate permutation tests and restricting gene list based on the fold change threshold with their default values (i.e., 10,000 and 2, respectively). All of the identified DEGs were stored for the next stage (i.e., prioritization of DEGs) as test group. Furthermore, the volcano plot and box plot of the imported data were demonstrated for each stage versus the normal samples.

### Prioritization for DEGs

To prioritize identified DEGs from the previous section using the evidence of the literature, GeneCards^17^ and ToPPGene^18^ websites were used, respectively. The GeneCards database site (i.e., https://genecards.org) was used to extract the literature evidence on reported genes (denoted by the train group) for a specific disease by using approximately 150 web sources and the keywords. For this purpose, the used keywords included < “breast cancer” + ”stage I” > , < “breast cancer” + ”stage II” > , < “breast cancer” + ”stage III” > , < “breast cancer” + ”stage IV” > , as well as inclusion of the results of all four stages. Then, the ToPPGene website (i.e., https://toppgene.cchmc.org), which used the functional annotation and protein interactions to prioritize the imported gene list, was used to order the test group of genes based on the train group to determine the most significant DEGs in all stages of breast cancer with the p-value less than 0.05. Moreover, the ToPPGene website uses the similarity scores of the train group based on fuzzy and Pearson correlation measurement values to score and rank the test group.

### Gene ontology, pathway and functional enrichment analyses of prioritized DEGs

To determine the biological and molecular functional processes of the prioritized gene list as well as their significant enriched pathways, the online tool provided in the DAVID v. 6.8 (Database for Annotation, Visualization, and Integrated Discovery) website (i.e., https://david.abcc.ncifcrf.gov/summary.jsp)^19,20^ was applied. This website took the advantages of the gene ontology (GO) annotation analysis and the Kyoto Encyclopedia of Genes and Genomes (KEGG) to cover the required properties. Moreover, the results with the p-value ≤ 0.05 were considered significant.

### Protein–protein interaction (PPI) network construction

The protein–protein interaction network among prioritized DEGs was constructed by the Search Tool for the Retrieval of Interacting Genes/Proteins (STRING database ver. 11 plugin^21^ for Cytoscape v.3.7.1^22^). The current STRING database (since January 19, 2019) contains 24,584,628 proteins from 5,090 organisms with 3,123,056,667 interactions. Moreover, the STRING database is experimentally dependent on BIND, DIP, GRID, HPRD, IntAct, MINT, and PID, and the cumulative information is extracted from curated websites Biocarta, BioCyc, GO, KEGG, and Reactome^21^. During the gene list import using the Cytoscape software, the confidence score cutoff value was set as 0.4 for PPI network construction and visualization. In the PPI network, the involved proteins are denoted by nodes, and their corresponding protein–protein interactions are presented as edges. To further investigate the PPI network of each of the breast cancer stages, the module (hub gene) analysis was performed using ClusterOne v.1.0 cytoscape plugin^23^ with its default values. Then, the significant modules with the p-value ≤ 0.05 were retrieved for biomarker identification. A protein with the highest connectivity degree in each candidate module will be considered a biomarker.

### Validation of gene biomarkers

To validate the identified gene biomarkers for each stage, three validation approaches were considered. These include (i) the Kaplan–Meier (KM) plotter tool, (ii) classification model development and validation, and (iii) literature search for the identified gene biomarkers.

#### Kaplan–Meier plotter tool

To further validate the prognostic value of the gene biomarkers obtained from the hub genes of five groups, the free online Kaplan–Meier (KM) plotter tool was used^24,25^. Using the KM plotter tool, a meta-analysis based approach on thirty-five separate datasets was presented to assess the gene biomarkers in terms of various survival rates such as relapse free survival (RFS) and overall survival (OS). However, it has been reported that there is no significant difference between recurrence or relapse or disease free survival and overall survival rates^26,27^. To this end, the relapse free survival (RFS) (n = 3,955) was used by restricting the analysis to only HER2 (ERBB2) considering the HER2 nature of the three abovementioned datasets. Moreover, to generate high-resolution images, an option, namely “Generate high resolution TIFF file” was enabled before drawing the Kaplan–Meier plot and then, their *p-*values were recorded for target biomarkers. Additionally, by analyzing the RFS rate, the clinical outcomes of a disease would be measured if the time to death of the patient would be observed rather than validating the prognostic value of the gene biomarkers at particular stages of a disease.

#### Classification model development and validation

To validate the prognostic value of the identified biomarkers for a specific disease, a non-linear classification model was developed. For this purpose, nine classification models in Orange 3.22.0, including support vector machine, k-nearest neighbors, stochastic gradient descent, random forest, artificial neural network, Naïve Bayes, logistic regression, CN2 rule inducer, and adaboost were considered^28^. Furthermore, cross-validated accuracy (CA), precision (positive predictive value), recall (sensitivity), F1 score (a harmonic mean of sensitivity), and AUC (area under curve) were assessed using validation criteria such as *k*-fold cross-validation (*k* = *5, 10),* LOOV (leave-one-out validation) as well as testing the model on train and test sets. Overall, the developed models would be validated both internally and externally.

1 $$Accuracy=\frac{TP+TN}{TP+TN+FP+FN}$$

2 $$ Precision=\frac{TP}{TP+FP} $$

3 $$ Recall=\frac{TP}{TP+FN} $$

4 $$ F1\,Score= \frac{2TP}{2TP+FP+FN} $$

#### Literature screening for potential genes

Another way of validating the identified genes was carried out based on the frequent appearance of the reported genes through experimental wet-labs of the literature investigations for the disease.

## Results

### Data preprocessing

The numbers of genes remained after applying the filtering criteria at stages I (normal:10, stage I:20), II (normal:10, stage II:80), III (normal:10, stage III:15), IV (normal:10, stage IV:141), and in the integrated group (normal:10, all samples at stage I, II, III, and IV:256) were 1,873, 2,034, 2,016, 2,279, and 2,471, respectively. Among the filtered genes, 832 (341 downregulated genes and 491 upregulated genes), 836 (392 downregulated genes and 444 upregulated genes), 980 (444 downregulated genes and 536 upregulated genes), 731 (455 downregulated genes and 276 upregulated genes), and 735 (464 downregulated genes and 271 upregulated genes) DEGs were identified using the two-sample t-test for the order of the abovementioned stages.

### Prioritization of DEGs

After searching the GeneCards database for the specified breast cancer terms, 2,264, 1,611, 1,856, 855, and 6,586 DEGs for stages I, II, III, IV, and the integrated group were extracted and exported as a .csv file and were set as training datasets for five groups, separately. Moreover, the identified DEGs for five groups from BRB-ArrayTools were set as test datasets. Then, the ToppGene database ranked the input test datasets based on training datasets in five groups separately for each stage. Considering the threshold of the p-value < 0.05, the numbers of the selected DEGs for the above order of stages were 287, 339, 365, 347, and 224 that could play an important role in five specified stages of breast cancer. Among those DEGs identified for stage I, 131 genes were downregulated and 156 genes were upregulated. The values of downregulated and upregulated genes for stages II, III, IV and all stage were 174 and 165, 176 and 189, 218 and 129 as well as 134 and 90, respectively. Table 1 presents the list of the top 10 upregulated and downregulated genes ranked for all stages considering their low *p*-values.

**Table 1:** Top 10 ranked genes resulted from ToppGene website based on significant p-values.

Rank	Gene symbol	Gene name	Expression	Overall *p-*value
**Stage I**				
1	CDK5	cyclin dependent kinase 5	Downregulated	7.44E−04
2	PSEN2	presenilin 2	Downregulated	9.04E−04
3	IKBKB	inhibitor of nuclear factor kappa B kinase subunit beta	Downregulated	9.11E−04
4	PRNP	prion protein	Upregulated	0.001222546
5	ITGB4	integrin subunit beta 4	Upregulated	0.001326232
6	DDX58	DExD/H-box helicase 58	Downregulated	0.001337916
7	BIN1	bridging integrator 1	Upregulated	0.001404577
8	SPRY2	sprouty RTK signaling antagonist 2	Upregulated	0.001584877
9	PYCARD	PYD and CARD domain containing	Downregulated	0.001615065
10	EDNRB	endothelin receptor type B	Upregulated	0.002032487
**Stage II**				
1	CDK5	cyclin dependent kinase 5	Downregulated	6.37E−04
2	FN1	fibronectin 1	Downregulated	6.57E−04
3	PRKCD	protein kinase C delta	Downregulated	7.17E−04
4	ADRB2	adrenoceptor beta 2	Upregulated	8.56E−04
5	PRNP	prion protein	Upregulated	9.66E−04
6	ITGB4	integrin subunit beta 4	Upregulated	0.001021494
7	DDX58	DExD/H-box helicase 58	Downregulated	0.001177986
8	NTRK2	neurotrophic receptor tyrosine kinase 2	Upregulated	0.001327566
9	PYCARD	PYD and CARD domain containing	Downregulated	0.001328362
10	TFRC	transferrin receptor	Downregulated	0.001379303
**Stage III**				
1	PRKCD	protein kinase C delta	Downregulated	6.06E−04
2	CDK5	cyclin dependent kinase 5	Downregulated	6.15E−04
3	PSEN2	presenilin 2	Downregulated	6.84E−04
4	IKBKB	inhibitor of nuclear factor kappa B kinase subunit beta	Downregulated	8.94E−04
5	ITGB4	integrin subunit beta 4	Upregulated	0.001086698
6	FOS	Fos proto-oncogene, AP-1 transcription factor subunit	Upregulated	0.001097674
7	BMPR1A	bone morphogenetic protein receptor type 1A	Upregulated	0.001143067
8	ATP1A2	ATPase Na + /K + transporting subunit alpha 2	Upregulated	0.001219288
9	GSN	gelsolin	Upregulated	0.001295466
10	TCF7L2	transcription factor 7 like 2	Upregulated	0.001296722
**Stage IV**				
1	APP	amyloid beta precursor protein	Downregulated	3.64E−04
2	CAV1	caveolin 1	Downregulated	3.81E−04
3	GNAS	GNAS complex locus	Upregulated	3.87E−04
4	PRKCD	protein kinase C delta	Upregulated	4.09E−04
5	CDK5	cyclin dependent kinase 5	Upregulated	4.77E−04
6	FYN	FYN proto-oncogene, Src family tyrosine kinase	Downregulated	7.47E−04
7	NR3C1	nuclear receptor subfamily 3 group C member 1	Downregulated	7.89E−04
8	STAT1	signal transducer and activator of transcription 1	Upregulated	7.92E−04
9	FLNA	filamin A	Downregulated	8.61E−04
10	IRS1	insulin receptor substrate 1	Downregulated	8.68E−04
**Integrated group**				
1	PRKCD	protein kinase C delta	Upregulated	5.23E−04
2	CDK5	cyclin dependent kinase 5	Upregulated	6.15E−04
3	PSEN2	presenilin 2	Upregulated	9.13E−04
4	ITGB4	integrin subunit beta 4	Downregulated	0.001097106
5	DDX3X	DEAD-box helicase 3, X-linked	Downregulated	0.001227256
6	DDX58	DExD/H-box helicase 58	Upregulated	0.001230075
7	MAPK9	mitogen-activated protein kinase 9	Upregulated	0.002138409
8	FKBP4	FK506 binding protein 4	Upregulated	0.002241011
9	LMNB1	lamin B1	Upregulated	0.00228287
10	DST	dystonin	Downregulated	0.002355127

### GO enrichment and KEGG pathway analysis

**Figure 2:**
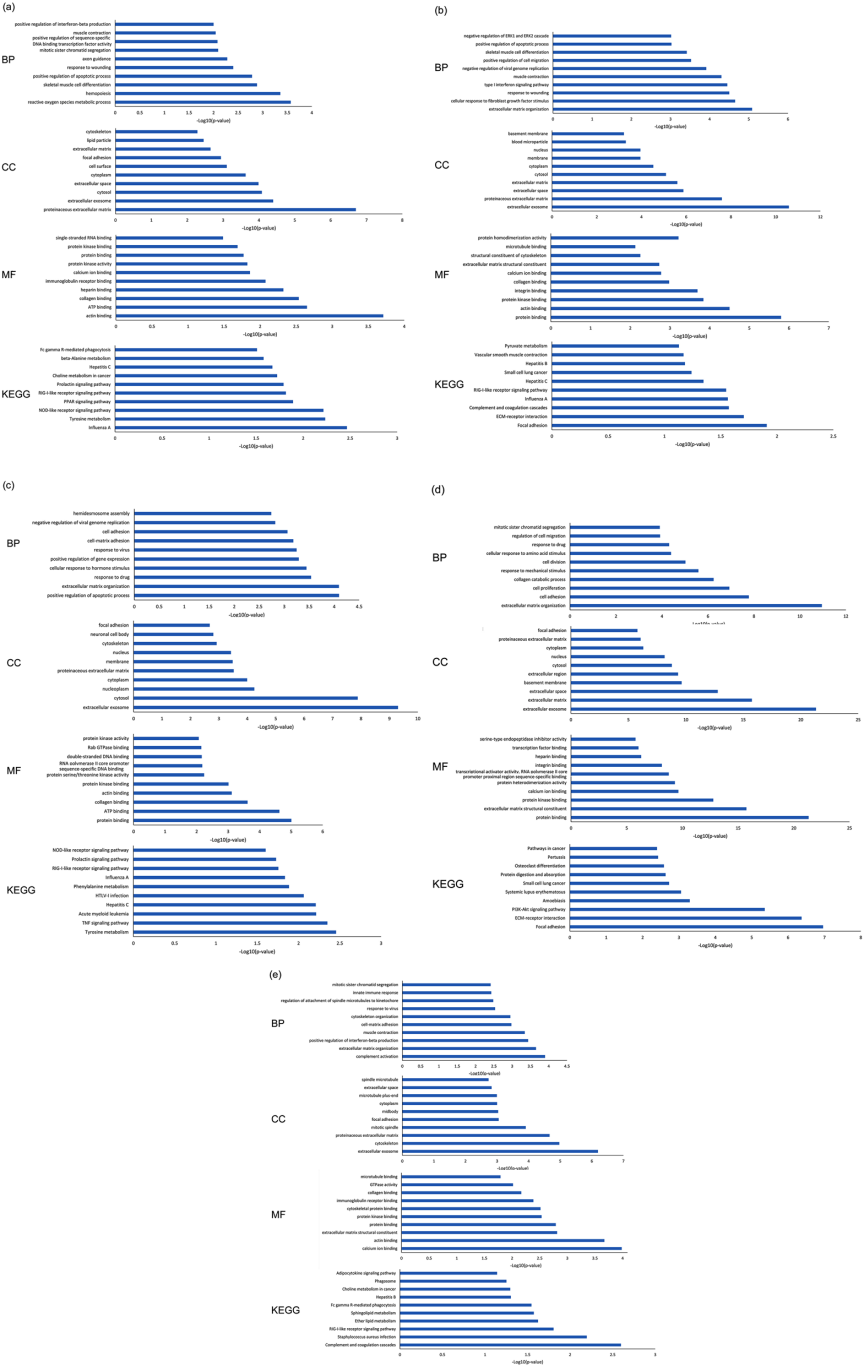
The biological processes (BP), cellular components (CC), and molecular functions (MF) for GO analysis as well as the KEGG pathway assessment for (**a**) stage I, (**b**) stage II, (**c**) stage III, (**d**) stage IV, and (**e**) Integrated group.

The output of the DAVID bioinformatics tool provides diverse biological and functional analyses on the prioritized genes in five groups. These include biological processes (BP), cellular components (CC), and molecular functions (MF) for GO analysis as well as the KEGG pathway assessment. Considering stage I, several biological processes (e.g., reactive oxygen species metabolic process, hemopoiesis), cellular components (e.g., proteinaceous extracellular matrix, extracellular exosome), molecular functions (e.g., actin binding, ATP binding), and KEGG pathways (e.g., Influenza A, Tyrosine metabolism) are mainly enriched by DEGs (Fig. 2a). Moreover, the DEGs at stage II are associated with extracellular matrix organization and cellular response to fibroblast growth factor stimulus in terms of BP, with extracellular exosome and proteinaceous extracellular matrix in terms of CC, with protein binding and actin binding in terms of MF as well as focal adhesion and ECM-receptor interaction in terms of KEGG pathways (Fig. 2b). The key genes at stage III are enriched in BP related to the positive regulation of the apoptotic process and extracellular matrix organization, in CC related to extracellular exosome and cytosol, in MF related to protein binding and ATP binding, and in KEGG pathways related to Tyrosine metabolism and TNF signaling pathway (Fig. 2c). Additionally, at stage IV, extracellular matrix organization, extracellular exosome, protein binding, and focal adhesion are the most statistically significant enrichments in BP, CC, MF groups and KEGG pathways (Fig. 2d). The GO analysis results of the integrated group show that DEGs in groups BP, CC, MF are significantly enriched in complement activation, extracellular exosome, and calcium ion binding. Furthermore, the KEGG pathways analysis for all stages reveals that complement and coagulation cascades and Staphylococcus aureus infection are significantly enriched by prioritized DEGs (Fig. 2e).

### PPI network analysis and hub genes identification

Using the Cytoscape and STRING database plugin, PPI networks are constructed for five groups (i.e., stage I (284 nodes and 512 edges), stage II (338 nodes and 1,263 edges), stage III (363 nodes and 1,170 edges), stage IV (346 nodes and 1909 edges), and the integrated group (221 nodes and 519 edges)). Among genes with higher interconnectivity within the constructed PPI networks of five groups, SMC4 (degree = 24, downregulated), FN1 (degree = 50, downregulated), FOS (degree = 42, upregulated), JUN (degree = 69, downregulated), and KIF11 and RACGAP1 (degree = 27, upregulated) for stage I, stage II, stage III, stage IV, and all stages, respectively, have the highest connectivity degrees in their PPI networks.

The significant outcomes for the ClusterOne module analysis in Cytoscape (p-value < 0.05) reveal 14, 9, 8, 4, and 6 protein modules for stages I, II, III, IV, and the integrated group, respectively.

### Verification of central gene biomarkers

#### KM plotter tool

According to the visualization and numerical results obtained from the KM plotter and analysis tool, it has been revealed that 33 out of 53 potential biomarkers have a statistical significant association with the recurrence of free survival for five groups in HER2 breast cancer. Table 2 lists the characteristics of each of 53 genes in terms of their stages, gene symbol and expression, and overall p-value.

**Table 2:** A summarized list of results of Kaplan–Meier plot tool for 53 potential genes categorized based on their stages and literature screening references.

Stages	Rank	Gene symbol	Expression	Overall P value	Related cancers	References
Stage I	1	SMC4	Downregulated	1.7e−14	ER-positive and ER-negative breast cancer	^38^
	2	IRF7	Downregulated	0.1861	Suppressor of an innate immune pathway in breast cancer	^38,39^
	3	POSTN	Downregulated	0.3289	A factor in preventing and treating breast cancer	^38,40^
	4	ABAT	Downregulated	8.9e−16	ER-positive and ER-negative breast cancer	^41^
	5	LMOD1	Upregulated	0.1821	Involved in the development of breast cancer	^42^
	6	TRIM2	Upregulated	0.7228	Invasive and basal-like breast cancer	^43,44^
	7	CHRDL1	Upregulated	2.4e−8	Malignant breast cancer	^45^
	8	MFGE8	Upregulated	0.1294	Triple-negative and ER + breast cancers	^46,47^
	9	GLRX5	Downregulated	0.0001	Breast cancer Neurological disorders such as Parkinson’s disease and those associated with ageing	^48^
	10	ELF5	Upregulated	0.1522	TNM staging system for all types of breast cancer and metastasis in breast cancer	^49,50^
	11	CSN2	Upregulated	1.0e−8	Invasive breast cancer triple-negative breast cancer	^51,52^
	12	PRLR	Downregulated	7.7e−5	Progression of breast carcinoma	^53–55^
	13	PPAP2B	Upregulated	9.4e−10	Coronary artery disease Breast cancer Tumor growth in breast cancer	^56–59^
	14	FZD2	Downregulated	3.3e−11	Breast cancer	^60–62^
	15	FZD7	Upregulated	0.6871	Breast cancer	^60–62^
	16	GPC4	Downregulated	0.0004	In both MCF-7 (human breast adenocarcinoma) and MCF-10F (normal-like breast cancer)	^63–65^
	17	CERS2	Downregulated	0.2238	Less invasive breast cancer	^66–68^
	18	UGCG	Downregulated	1.1e−10	Triple-negative BC ER-negative BC tumors Lung metastases	^69–73^
	19	LIPE	Upregulated	0.0051	Prognostic cofactor in BC Cancer lipolysis	^74–76^
	20	PLIN1	Upregulated	2.6e−5	HER2 tumors Breast cancer Triple-negative breast cancer	^77–79^
Stage II	1	CCNB2	Downregulated	< 1e−16	Basal-like, HER2, and luminal breast cancers	^80–83^
	2	OAS3	Downregulated	0.697	Mutated gene in breast cancer	^84–86^
	3	IRF7	Downregulated	0.1861	Suppressor of an innate immune pathway in breast cancer	^38,39^
	4	OAS1	Downregulated	0.5676	Development of various cancer types like breast cancer	^85–87^
	5	CDKN1C	Upregulated	1.9e−5	Breast tumors	^88,89^
	6	PEG3	Upregulated	0.0029	Several cancers such as breast and ovary cancers	^90,91^
	7	PHLDA2	Downregulated	4.0e−10	PRL treatment Tumor progression	^92,93^
	8	PLAGL1	Upregulated	0.3823	Breast cancer patients under radiotherapy treatment	^94^
	9	SGCE	Upregulated	0.0293	Progression of breast cancer invasion in terms of stromal changes	^95^
	10	SLC22A18	Downregulated	3.2e−8	Breast cancer	^96^
	11	SERPING1	Upregulated	1.3e−8	Breast carcinoma cells	^97^
	12	ACTA2	Upregulated	0.6126	Metastasis of breast cancer cells Dimerization of epidermal growth factor receptor (EGFR) and HER2	^98–100^
	13	LCP2	Downregulated	0.7828	Predicting the development of secondary lymphedema followed by breast cancer surgery	^101,102^
	14	ABCG1	Downregulated	0.2418	High expression level of ABCG1 transporters in MCF-7 cells	^103,104^
	15	ZFP36L1	Upregulated	0.0507	In all types of breast cancer	^105^
	16	BICC1	Upregulated	1.0e−10	Cystic renal dysplasia embryonic node, kidney, liver, and pancreas in the mouse Basal-like breast tumors	^106,107^
	17	SSPN	Upregulated	0.0007	Several types of cancer, including breast invasive cancer	^108–110^
Stage III	1	FEN1	Downregulated	< 1e−16	High stages of breast cancer Inhibition of the tumor growth	^111–113^
	2	ADH1B	Upregulated	0.0068	Risk factors for breast cancer	^114–116^
	3	IRF7	Downregulated	0.1861	Suppressor of an innate immune pathway in breast cancer	^38,39^
	4	ACTA2	Upregulated	0.6126	Metastasis of breast cancer cells Dimerization of epidermal growth factor receptor (EGFR) and HER2	^98–100^
	5	CLDN5	Upregulated	9.4e−6	In both breast tumor stromal (BTS) and prostate tumor stromal (PTS)	^117,118^
	6	SLC31A1	Downregulated	0.4854	Progression of breast cancer	^119,120^
	7	FBLN1	Upregulated	3.6e−5	In several types of cancer, including breast cancer	^121,122^
	8	MFAP4	Upregulated	5.8e−9	In cell adhesion, motility, invasion, and metastasis of BC	^95,123,124^
	9	COL1A2	Downregulated	0.4121	High expression level at higher stages of breast cancer	^125–127^
	10	ASPN	Downregulated	0.2608	Upregulated expression in breast cancer	^128,129^
Stage IV	1	NUSAP1	Upregulated	< 1e−16	A potential biomarker clinically correlated with breast cancer	^130,131^
	2	COL6A2	Downregulated	0.0038	Important role in breast cancer development	^132,133^
	3	HIST1H2BD	Upregulated	0.2745	ER-positive breast cancer In breast cancer development	^42,134^
	4	HIST1H2BH	Upregulated	0.0006	ER-positive breast cancer In breast cancer development	^42,134^
	5	HIST1H2BK	Upregulated	8.6e−8	ER-positive breast cancer In breast cancer development	^42,134^
	6	HIST2H2BE	Upregulated	0.1077	ER-positive breast cancer In breast cancer development	^42,134^
Integrated group	1	KIF11	Upregulated	< 1e−16	Triple-negative breast cancer	^135,136^
	2	IRF7	Upregulated	0.1861	Suppressor of an innate immune pathway in breast cancer	^38,39^
	3	OAS1	Downregulated	0.5676	Development of various cancer types like breast cancer	^85–87^
	4	OAS3	Downregulated	0.697	Mutated gene in breast cancer	^84–86^
	5	SGCE	Upregulated	0.0293	Progression of breast cancer invasion in terms of stromal changes	^95^
	6	ALDH7A1	Downregulated	0.0208	Breast cancer Potent marker in different types of cancer like prostate cancer	^137–139^
	7	ABCG1	Downregulated	0.2418	Breast cancer	^140^
	8	C1S	Downregulated	1.2e−6	HER2-positive and basal-like breast cancer	^140^

#### Performance of nine classifiers

The classification prediction results of all nine non-linear models (i.e., AUC, CA, F1 score, precision, and recall parameters) were investigated. In the *k*-fold cross-validation procedure to keep and possibly increase the stability of the models within the folds, the stratification sampling is used. Except, the performance of the models on the test set, almost all of the machine learning classifiers are trained and cross-validated at the highest values while considering the five-fold cross validation, ten-fold cross validation, stratified shuffle sampling trained on 66% of data, leave one out validation, and trained and tested on the whole dataset. Once the trained model is tested on the test set, the performance results for stages I, II, and III show that naïve Bayes, random forest, and naïve Bayes outperform the other classifiers with 0.87, 0.83, and 0.89 AUC values, respectively. The results are indicative of the fact that the computational classification models are capable of validating the identified genes from the systems biology approach for several stages of breast cancer.

#### Literature screening for identified genes

The other tactic commonly used in the systems biology related studies for validating the identified genes from a specific computational methodology is to gather the required evidence from the literature reports on a specific determined gene in a known disease (i.e., breast cancer). To this end, searching results present that all of the fifty three genes are found to be responsible for cell proliferation, growth, motility, and development at several stages of breast cancer disease. The next section discusses detailed information on these genes (Table 2).

## Discussion

Breast cancer as a heterogeneous disease and the most common invasive cancer is the second leading cause of mortality among women globally^29^. During the last thirty years, the trend of mortality rate for breast cancer in developed countries has been dramatically decreased; however, the condition for low-income countries has no significant changes^30^. The success in the mortality rate reduction of breast cancer in high-income countries is mostly owing to the improved treatment and early stage diagnosis as well as the appropriate selection and administration of therapies^30^. This will be followed by prolonging RFS and OS without complications^29^.

In this research, three microarray datasets, including stages I, II, III, IV, and the integrated group, were used, preprocessed, normalized and analyzed from which the significant DEGs for five groups were identified. After that, they were ranked based on the literature involved genes in breast cancer and selected based on the statistical significant p-value < 0.05. Then, GO and KEGG pathways analyses as well as PPI network construction were performed. The biological processes (BP), cellular components (CC), and molecular functions (MF) were also assessed for enrichment pathways. Moreover, the PPI network analysis using the STRING database revealed several effective hub genes for five groups separately. The significant gene biomarkers with the highest connectivity degree within the hub genes were selected. The validation of the obtained gene biomarkers in terms of recurrence free survival rate in HER2 was statistically carried out by Kaplan–Meier plotter tool with p-values less than 0.05. Moreover, the internal and external validation procedures revealed that the machine learning classification models specifically those developed based on naïve Bayes and random forest by employing various biomarkers at several stages were successful in differentiating between stages and normal samples with good predictive power. Finally, in Table 2, the available evidences collected from the experimental literature reported for breast cancer has been retrieved and listed according to the identified gene biomarkers. Additionally, some of the identified biomarkers were found to be common among different TNM stages. For example, IRF7 was the significant biomarker for stages I, II, and III; and, ACTA2 biomarker was found to have an increasing expression across stages II and III.

According to the outcomes of the current study, we identified a signature of potential biomarkers for BC stages to specifically diagnose breast cancer at developed stages as well as very early stages. These biomarkers could potentially be the target of wet-lab researchers for future investigations. The mathematical models developed for BC prediction and diagnosis at various stages showed significantly high and reasonable performance in clinical outcomes employing the identified biomarkers. It is worth noting that the current study is conducted for the first time that studied the high throughput gene profiling datasets for four stages of BC as well as its integrated stage. Finally, the strong point of the study relied on the three validation methodologies, however, the Kaplan–Meier analysis did not find some of the biomarkers statistically significant.

The systems biology approach could enlighten the path for wet-lab investigators in rapid identification of stages in patients with BC. Moreover, the developed non-linear models could be utilized in prediction procedure after the gene expression values for target biomarkers are determined through experimental tests. The workflow of the current study could be applied for other future microarray studies in terms of involving and investigating the stages of the diseases. Furthermore, the identified biomarkers along with their involved signaling pathways could be beneficial for drug design and discovery agents considering various disease stages and hence, the disease could be controlled, managed and treated at very early stages.

Any researches specifically those carried out on systems biology approaches will have limitations and it seems to be normal. Due to the computational nature of these studies, there will remain gaps between the wet- and dry-labs for further validating the results. The experimental and clinical literature studies do only report on the genes involved in BC disease without stating their stages. The lack of available sufficient microarray datasets in the repository databases investigating the stages of BC made us consider the stages and grades of BC equivalent for the validation process.

During the last decades, extensive genome-wide association studies and next generation sequencing techniques were conducted and applied to identify the potent biomarkers using bioinformatics and experimental approaches for various diseases such as Parkinson’s disease and prostate cancer considering the exponential growth of Big Data generation in the field^31–35^. For future researches, it is useful to investigate the genome-based studies in a centralized manner to provide the datasets in further details in terms of being more specific at the disease stages and the follow-up procedures. Moreover, owing to the large generation of genome datasets, handling and managing them computationally and experimentally are still of many researches’ interest in the world. Therefore, close cooperation among systems biologists, bioinformaticians, and biologists is required in to identify potential biomarkers and their involvement in signaling pathways. In other words, understanding the functions of the target signaling pathways in specific diseases is highly important in accelerating the development of new experimental drugs and diagnostics, paving the ways for personalized medicine and improving translational sciences^32,36,37^.

## Conclusions

In this study, three HER2-negative breast cancer datasets were analyzed to identify differentially expressed genes and construct protein–protein interaction networks as well as GO enrichment and KEGG pathway analyses for the TNM-based staging system. The results indicate that a 53-gene signature is responsible for breast cancer prognosis at various stages. The identified gene signature could be further utilized in personalizing medicine for individuals with breast cancer. The identified PPI modules significantly involved at different stages of breast cancer show a different number of connectivity ranging from 1 to 69. The interesting finding noticeable in the results is that the lower number of interactions within hub genes is not correlated with the importance of genes as potential biomarkers. For example, module 5 with only three genes and two connections shows significant expression (downregulation) in the integrated group. Her2-negative breast cancer was further confirmed by the literature reports. Moreover, the Kaplan–Meier tool for assessing the recurrence-free survival rate is not a measure to exclude a biomarker based only on its statistical significant *p-*value. For instance, in Table 2, there are 20 genes identified to be non-significant in the RFS rate assessment evaluated by the KM tool. However, for example, IRF7 identified as a biomarker for almost all groups has not been significantly related to the RFS rate. However, according to the literature, IRF7 is significantly correlated with breast cancer development. Therefore, non-significant p-value in the KM assessment does not decrease the importance of an identified biomarker. The outcomes of this research have paved the way to evaluate the status of breast cancer development in terms of the TNM-based staging system. All of the identified DEGs were involved in breast cancer as confirmed by the evidence available in the literature derived solely from experimental studies. What is missing from the clinical data in the literature is the staging of the condition, which now can be answered using the panel of gene biomarkers proposed in this study.
